# Influence of high porous sponges for improving the interfacial evaporation from hemispherical solar distillers

**DOI:** 10.1038/s41598-023-44137-z

**Published:** 2023-10-11

**Authors:** Ravishankar Sathyamurthy, A. E. Kabeel, Mohammed El Hadi Attia, Mohamed Abdelgaied, A. S. Abdullah, Kuma Gowwomsa Erko

**Affiliations:** 1 Department of Mechanical Engineering, King Fahd University of Petroleum and Minerals, Dhahran, Dammam, Saudi Arabia; 2 IRC-Renewable Energy and Power Systems, King Fahd University of Petroleum and Minerals, Dhahran, Dammam, Saudi Arabia; 3 Department of Mechanical Engineering, Islamic University of Madinah, Medina, Saudi Arabia; 4 Mechanical and Power Engineering Department, Faculty of Engineering, Tanta, Egypt; 5 Faculty of Engineering, Delta University for Science and Technology, Gamasa, Egypt; 6 Department of Physics, Faculty of Science, University of El Oued, 39000 El Oued, Algeria; 7 Mechanical Engineering Department, College of Engineering, Prince Sattam Bin Abdulaziz University, Al-Kharj, Saudi Arabia; 8 Faculty of Engineering, Tanta University, Tanta, Egypt; 9 Department of Mechanical Engineering, Ambo University, Ambo, Ethiopia

**Keywords:** Environmental sciences, Engineering, Energy infrastructure, Mechanical engineering

## Abstract

The present study aims to improve the palatable water production from the hemispherical cover solar distiller (HSD). To augment the palatable water produced from the hemispherical cover, a black sponge was utilized as a porous medium using different thicknesses, which augments the interfacial evaporation through the capillary effect of the water through the sponge. The rate of condensation of the hemispherical cover depends on the higher interaction of air from the ambient through wind velocity as the exposure area of the hemispherical cover is relatively higher as compared to the other traditional distillers. The rate of evaporation from the distillers depends on the interfacial materials used in the distillation unit, and this is achieved by using a highly porous black sponge to attain a higher evaporation rate. The thickness of the black porous sponge was optimized (1 to 4 cm), which was the operating parameter for better interfacial evaporation through the sponge, and the same has been compared to the conventional HSD without a porous sponge medium. Results showed a significant improvement in the evaporation rate using a porous medium as the palatable water produced from the HSD was improved by 72.29% using 3 cm as sponge thickness inside compared to the conventional HSD without the porous medium. The cumulative palatable water produced from the HSD using 3 cm as sponge thickness was found as 7150 mL/m^2^, whereas the conventional HSD without sponge, it was found as 4150 mL/m^2^. Moreover, using a porous sponge layer as an interfacial evaporation medium, the exergy and energy efficiencies were improved by about 512.87 and 70.53%, respectively. Similarly, with the influence of a porous sponge as an interfacial evaporation medium, the distilled water cost decreased by 41.67% more than the conventional HSD.

## Introduction

The world is moving in the twenty-first century towards the use of renewable energy (solar energy) to reduce pollution and have a clean environment^1–3^. Fresh water and energy are among the most important resources in the world, but the real problem is that both are not available in sufficient quantities^4–6^. Among the most important factors of water scarcity is the low groundwater level and pollution of the waters of rivers and valleys. Therefore, using solar energy to desalinate brackish water is the best solution to the scarcity of drinking water because this process is environmentally friendly^7–9^.

Rajaseenivasan et al.^10^ enhanced the still productivity by utilizing simple modifications (fins, sponges, and wicks). The results show that the productivity is 3.05 L/m^2^/day with the modifications. Velmurugan et al.^11^ augmented the potable water produced from the solar using fins (extended surfaces), sponges (porous medium), and wicks (capillary effect). It was reported that the extended surfaces on the traditional SS improved the potable water generation by 45.5%, whereas using porous medium and capillary effect through wick material showed a significant improvement of about 15.3 and 29.6%, respectively. Kalidasa et al.^12^ tested the influences of rectangular aluminum fin covered with different wicks on a double slope yield. The black cotton cloth was discovered to be more productive than the other wicks. Abu-Hijleh and Rababa'h^13^ employed sponge cubes to improve the still evaporation rate. They demonstrated that the distillate production rose by 18–273% compared to a traditional still without cubes sponge. Velmurugan et al.^14^ investigated stepped solar still and augmented the thermal performance and freshwater generation using various materials such as sponges, pebbles, and baffle plates to increase the area of surface water. They found that pebbles and sponges yielded the highest production of 100%, whereas pebbles and baffle plates yielded 78%. The influence of sponge densities varied from 16 to 30 kg/m^3^ on freshwater generation from the TSS was experimentally assessed by Abdelgaied et al.^15^. It was concluded that floating sponges on the absorber and sponge density of 16 kg/m^3^ produced the maximum freshwater of 5.92 L/m^2^/day, which was higher than the reference solar still (3.72 L/m^2^/day). The impact of pin fins as an extended surface and the influence of an external condenser for augmenting the palatable water produced from the TSS was experimentally analyzed by Abdelgaied et al.^16^. The results show that a cumulative yield for using the external condenser and fins reached 5.94 L/m^2^/day, with an improvement of 70.2% compared to the reference case. The effects of fins on solar still yield of potable water was experimentally investigated by Srivastava and Agrawal^17^. They conducted that the yield enhanced by 56% in February and 23% during the month of May over conventional still. The effects of hollow fins (circular and square) with wick materials on still production at various water depths were investigated by Rajaseenivasan and Srithar^18^. They found that the distillate yields reached 4.55 kg/m^2^/day in the distiller with fin and wick covering, whereas the traditional distiller yields 3.16 kg/m^2^/day. The influence of fins on the absorber of double slope SS for freshwater augmentation was experimentally assessed by Sadhana et al.^19^. The finned basin daily production is increased by about 18% higher than that of a conventional still. Kabeel et al.^20^ conducted the effects of cement-coated red bricks on distiller accumulative production of potable water. They concluded that the potable water produced from the modified thermal energy storage improved by about 45% more than the conventional distiller. Attia et al.^21^ examined the influences of increased graphite concentration on hemispherical distiller behavior. They found that yield increases with increasing concentration up to 35 g/L of graphite, but it stabilizes thereafter, although with increasing graphite concentrations. Attia et al.^22^ studied the effects of increased sand concentration on a hemispherical solar distiller performance. They found that the yield increases with increasing concentrations of up to 30 g/L of sand, but it starts decreasing with increasing sand concentrations of more than 30 g/L. Kabeel et al.^23^ examined the thermal performance of a hemispherical solar distiller at increased phosphate grain concentration. They found that yield increases with increasing concentration up to 30 g/L of phosphate grains, but it stabilizes thereafter, although with increasing phosphate grains concentrations. Attia et al.^24^ experimentally studied the impact of saltwater depths on the acrylic solar still with an extended surface. The highest cumulative potable water produced from the acrylic SS with fins was recorded as 5.67, 5.16, and 4.41 kg/m^2^ for saltwater depth varied from 1, 2, and 3 cm, respectively. Attia et al.^25^ used black gravel as sensible heat storage materials at different sizes (4 mm, 8 mm, 11 mm, and 16 mm) to increase the cumulative yield of hemispherical distillers. They conducted that for gravel sizes of 4, 8, 11, and 16 mm, and the hemispherical distillers yield with black gravel was 5.7 kg/m^2^, 6.45 kg/m^2^, 6.9 kg/m^2^, and 7.7 kg/m^2^, respectively, compared to 4.9 kg/m^2^/day for reference distiller. Attia et al.^26^ used iron fins inside the basin of a hemispherical solar distiller and studied the influence of the height of the fin and the spacing distance of the fin for improving thermal performance. The height of the fin varied from 1 to 3 cm, and the spacing between the fins in the absorber varied from 5 to 7 cm. For maximum freshwater production, the optimum spacing between the fins and the height of the fin was 7 and 2 cm, respectively. Attia et al.^27^ modified the hemispherical distiller performance via phosphate pellets with two concentrations (1 and 2%). The accumulated potable water yield reached 4.6, 6.15, and 6.85 L/m^2^ from the traditional hemispherical solar stills, and stills using 1 and 2% phosphate pellets, respectively.

Sampathkumar et al.^28^ conducted the impact of a mixture of beach sand with paraffin wax as hybrid storage materials on the yield of solar distillers. They found that the yield improved from 1820 to 2950 mL/m^2^ for using hybrid storage materials. Suraparaju and Natarajan^29^ improved the condensation rate of solar still using the natural fibers on the glass cover. They found that using the Jute Fibers improved the yield from 2.23 to 3.18 L/m^2^. Suraparaju and Natarajan^30^ improved the condensation rate of solar still using glass cooling and sisal fibers. They found that using glass cooling and sisal fibers improved the yield by 104.5% compared to classical solar distillers. Sahu et al.^31^ empirically improved the yield of solar distiller by 46% using PCM. Suraparaju et al.^32^ empirically studied the influences of paraffin wax on the yield of solar distiller. Singh^33^ studied the influences of evacuated annular tube collectors with parabolic solar receivers on the performance of double-slope solar distillers. Singh and Gautam^34^ and Singh and Samsher^35^ studied the influences of evacuated annular tube collectors with modified compound parabolic concentrators on the behavior of double-slope solar distillers. Singh and Samsher^36^ presented a comprehensive review of solar distillers incorporated with evacuated tube collectors. Singh and Samsher^37^ conducted the impact of incorporating the evacuated solar tube collector with compound parabolic concentrators with a solar desaltification unit. Singh et al.^38^ presented a comprehensive review of the different modifications that were conducted on the active solar distillation technology.

This study aims to increase freshwater produced from solar distiller devices to overcome the problem of freshwater shortages, especially in remote countries. where the major advantage of hemispherical solar distillers is a larger cover surface exposed to wind velocity compared to other traditional single slope solar distillers, which simultaneously reduces the cover temperature and leads to an effective rate of condensation. Therefore, the increase in the rates of evaporation inside the HSD represents an important axis to achieve the maximum benefit from the low temperature of the hemispherical cover and the increase in the productivity of the HSD from distilled water. To achieve this goal, a high porosity black sponge layer for improved interfacial evaporation was used to achieve the highest rates of evaporation and condensation within the hemispherical cover solar distillers. Based on the previous literature, it is found that the optimum density of the sponge material is 16 kg/m^3^, whereas the thickness of the porous medium is optimized in the present study. To get the optimal thickness of the porous sponge layers, the different thicknesses of porous sponge layers varied from 1 to 4 cm were used in the hemispherical distiller, studied, and compared to the reference HSD without porous sponge medium. To achieve this experimentally, three hemispherical distillers are manufactured and tested, the first being a reference unit without a sponge layer, while the other hemispherical distillers contain high porosity black sponge layers at different thicknesses. The experimentation was carried out in two stages.

Based on the research gap identified in the above studies and the aims of the present research work, the methodology of the present research work follows as design details, construction, and operation of the experimental test rig as shown in "Experimental methodology" section; a detailed explanation of the ruling equations for the performance of HSD as shown in "Thermal and exergy efficiency correlation" section; analysis and discussion of the recorded experimental data in detail as shown in "Results and discussions" section; an economic analysis of the statement of the economic feasibility of the proposed design of solar distillers as shown in "Economic analysis" section; and the most important results and the recommendations of future work as shown in "Conclusion and future recommendation" section.

## Experimental methodology

### Test-rig construction

**Figure 1:**
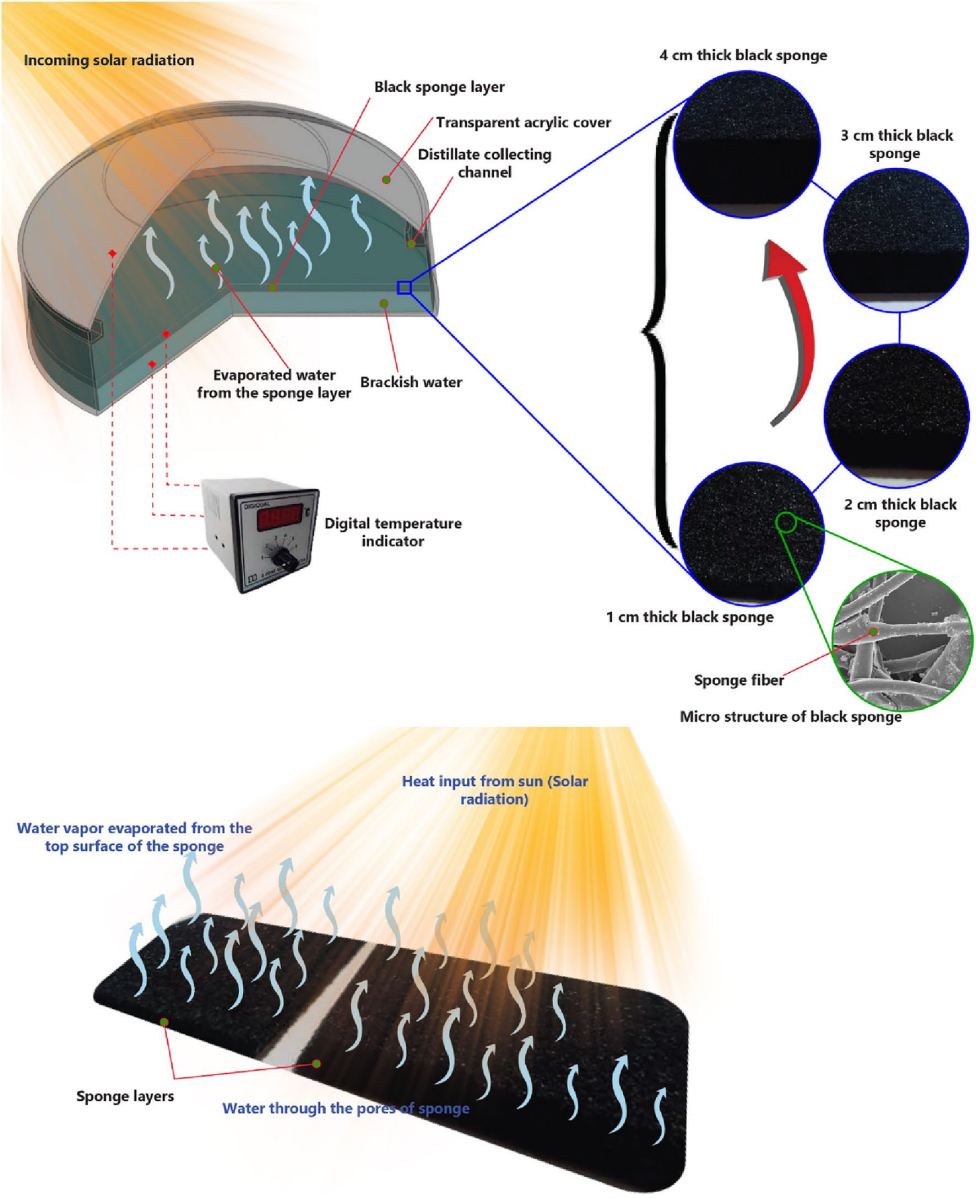
The schematic diagram of the hemispherical distiller.

The layout design of the hemispherical-basin distiller, as presented in Fig. 1, the hemispherical distiller consists of (a) a circular wooden basin of 50 mm thickness (360 mm diameter and 45 mm depth); (b) a Transparent cover hemispherical (400 mm diameter and 3 mm thick); and (c) a channel for collecting condensate water droplets. In addition, the inner basin surfaces of the distillers are coated with black silicone to obtain the maximum absorption of solar rays.

The present experiment aimed to achieve the highest hemispherical distiller's cumulative yield. The study aims to demonstrate the optimal thickness of high-porosity black sponge layers that provide the best interfacial evaporation rates within the hemispherical distiller. Compared to other typical solar distillers, hemispherical distillers have a higher surface area exposed to the wind, which concurrently lowers the cover temperature for an efficient condensation rate.

**Figure 2:**
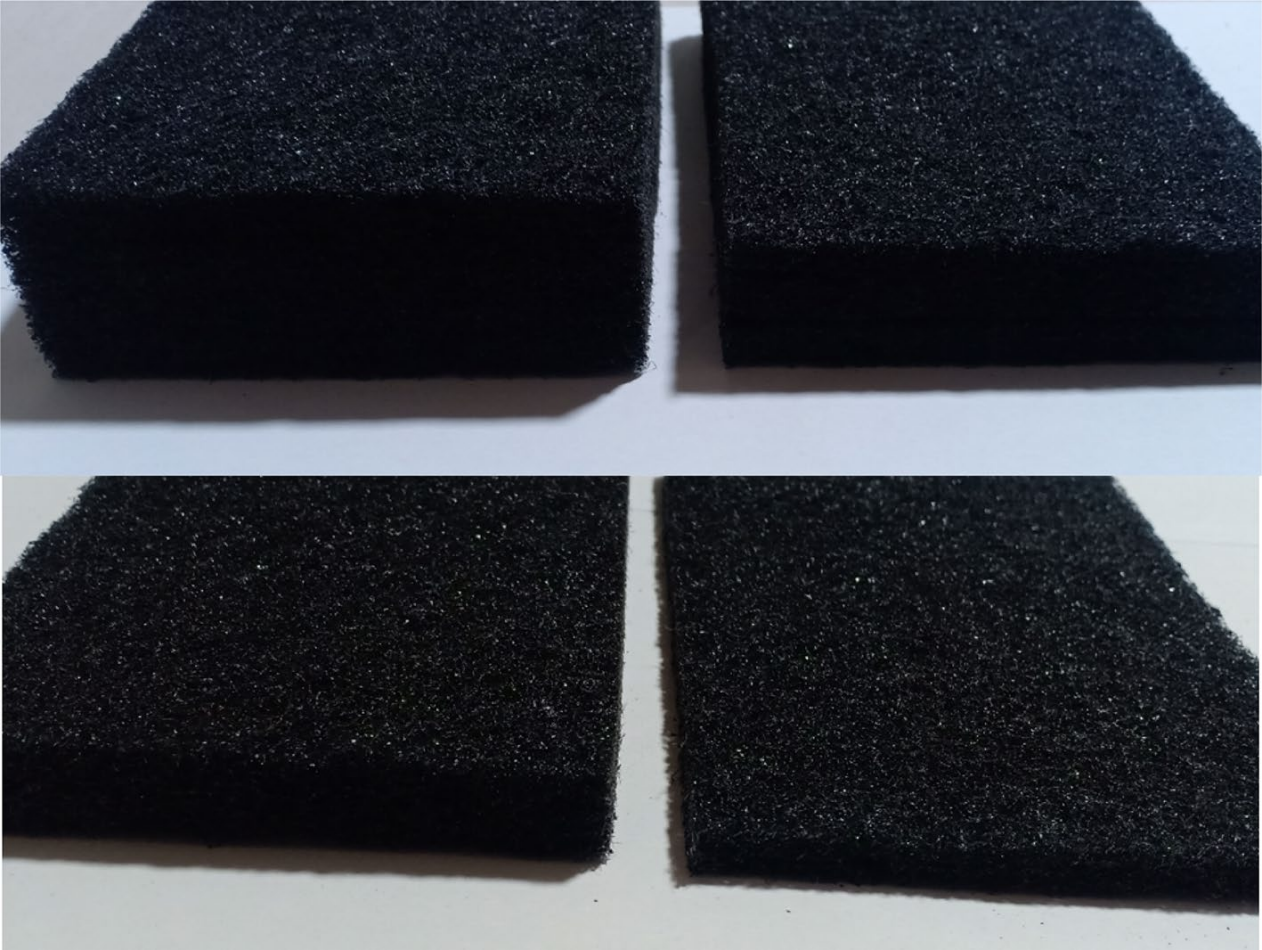
Image of high porosity black sponge layers at different thicknesses (4, 3, 2, and 1 cm).

**Figure 3:**
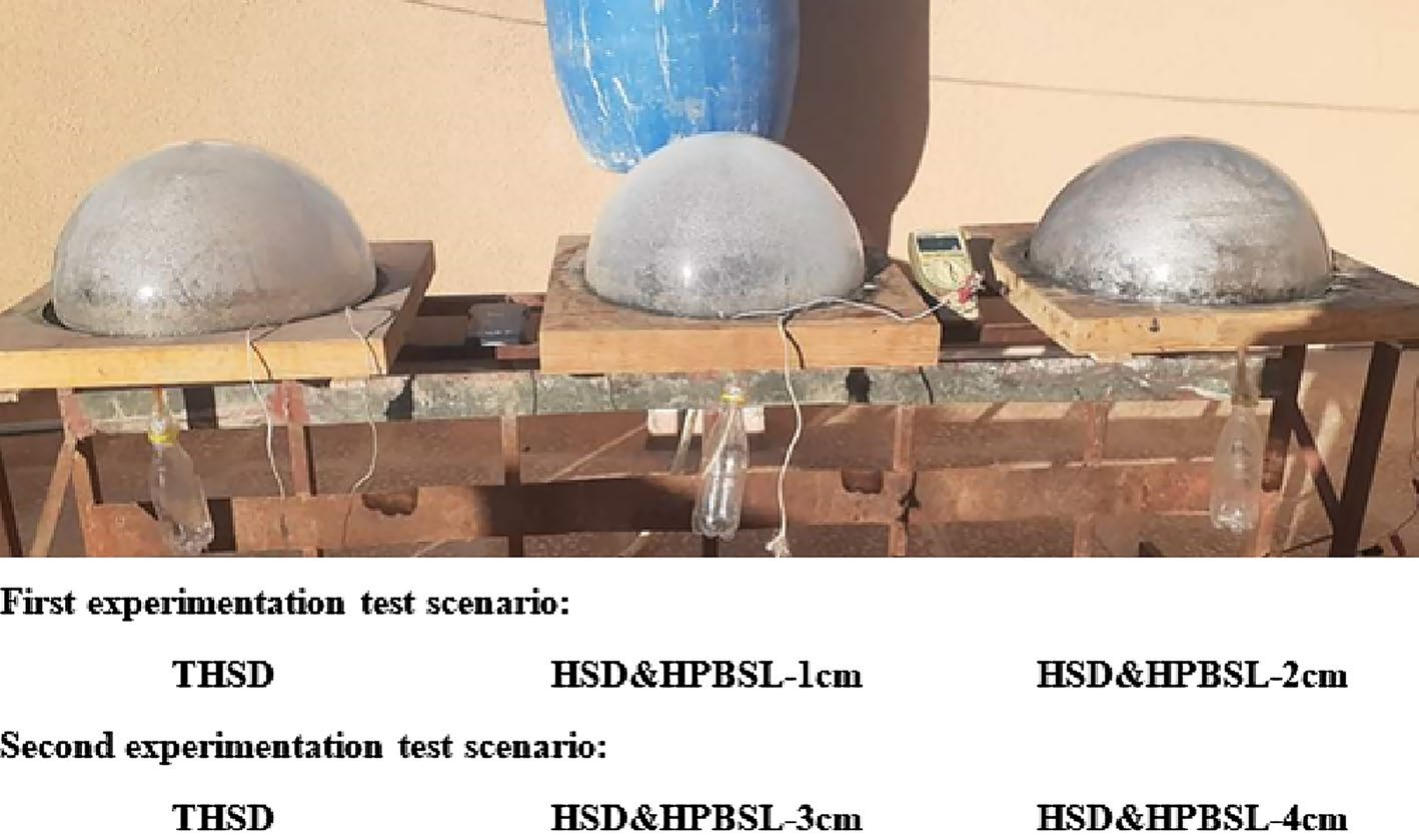
Photo of the experimental setup.

Therefore, a viable choice to obtain the largest evaporation and condensation rates from the hemispherical solar still is to incorporate a high porosity black sponge layer within the basin. An image of high-porosity black sponge layers at different thicknesses (4–1 cm) with the same density of 16 kg/m^3^ is shown in Fig. 2. The thickness of the black sponge is the only operating parameter to optimize, and the same has been compared to the SS without porous sponge as interfacial evaporation medium. The solar stills were fabricated and tested under the same weather and climatic conditions (Fig. 3). The dimensions used to fabricate the SS are illustrated above.

The first is a traditional hemispherical solar distiller (THSD), a reference distiller, while the other hemispherical solar distillers contain high porosity black sponge layers at different thicknesses (1–4 cm). Experiments were conducted in two different stages, namely: (i) two thicknesses of high porosity black sponge layer (1 and 2 cm) are fixed in second and third distillers (HSD & HPBSL—1 cm and HSD & HPBSL—2 cm) and compared to THSD. In the second stage, two thicknesses of high porosity black sponge layer (3 and 4 cm) are fixed in second and third distillers (HSD & HPBSL—3 cm and HSD & HPBSL—4 cm) and compared to THSD.

### Experimental procedures

The experimentation tests were conducted in two stages. In the first stage, two black sponges with higher porosity are used as layers (with thicknesses of 1 and 2 cm) in the second and third distillers (HSD & HPBSL—1 cm and HSD & HPBSL—2 cm), respectively, and compared to THSD. Two black sponges with higher porosity are used as layers (with thicknesses of 3 and 4 cm) and fixed in the second distiller and the third distillers (HSD & HPBSL—3 cm and HSD & HPBSL—4 cm), respectively, and compared to thermal performance and cumulative palatable water from THSD. During the experimentation test, the mass of basin water remained constant at 1.2 kg during the test period from 8:00 a.m. to 7:00 p.m. Each experimental test stage was compared under identical weather conditions on 10 and 11 May 2022, between 8:00 a.m. and 7:00 p.m.

To find out how different black sponge thicknesses affect the total yield of distillers. The thermocouples are inserted at different points of the distiller, such as the temperatures of the sponge layer, the temperature of the water, the inner and outer surface temperatures of the hemispherical cover, and the ambient temperature for the three hemispherical distillers. A solar power meter was utilized to record the sun rays in the test location. This equipment has a precision of 5.78 W/m^2^ for measuring sun intensity in the range of 0–1999 W/m^2^. A channel is affixed down the hemispherical cap to collect a condensed droplet that slips off the plastic hemispherical cover's inner surface and flows to the measuring jar. The hourly yield is measured in a glass beaker with a capacity of 500 mL. Table 1 shows the measuring instrument, measuring parameter, type, range, accuracy, and uncertainty. The uncertainties in the experimental recorded data and calculated results are calculated using the steps presented by Holman^39^. The uncertainties in the calculated daily cumulative distillate yield, exergy efficiency, and thermal efficiency are ± 2.13%, ± 2.37%, and ± 2.26%, respectively.

**Table 1:** Specifications of measuring instrumentations.

Instrument	Measuring parameter	Type	Accuracy	Range	Uncertainty
Thermocouple	Water, glass, ambient temperature	PT100 (RTD)	± 0.1 °C	− 100 to 1500 °C	1.13
Graduated cylinder	Palatable water	Borossil	± 1 mL	0–500 mL	2.45
Solar power meter	Solar radiation	TES1333	± 10 W/m^2^	0–2500 W/m^2^	3.76

## Thermal and exergy efficiency correlation

The daily thermal efficiency of the SS with and without porous sponge as interfacial evaporation medium is given as^40,41,42^,

1 $${\eta }_{th}=\frac{\sum \left(\frac{{\dot{m}}_{dw}}{3600} \times {h}_{fg}\right)}{\sum \left(I\left(t\right)\times {A}_{a}\right)}$$

where: ${\dot{m}}_{dw}$ is the yield (kg/h); ${A}_{a}$ is the absorber area (m^2^); I(t) is a solar radiation (W/m^2^); and ${h}_{fg}$ is latent heat (J/kg), which is calculated by Kabeel et al.^43^:

2 $${\mathrm{h}}_{fg}={10}^{3}\times \left[2501.9-\left(2.40706 {\mathrm{T}}_{bw}\right)+\left(1.192217\times {10}^{-3}{{\mathrm{ T}}_{bw}}^{2}\right)-\left(1.5863\times {10}^{-5} {{\mathrm{ T}}_{bw}}^{3}\right)\right]$$

Accumulated exergy efficiency ${\eta }_{ex}$ calculated as follows:

3 $${\eta }_{ex.}=\frac{\sum {Ex}_{out}}{\sum {Ex}_{in}}$$

Input exergy to a basin of hemispherical solar distiller ${Ex}_{in}$ calculated as^44^:

4 $${Ex}_{in}={A}_{a}\times I(t)\left[1-\frac{4}{3}\times \left(\frac{{T}_{a}+273.15}{6000}\right)+\frac{1}{3}\times {\left(\frac{{T}_{a}+273.15}{6000}\right)}^{4}\right]$$

Output exergy from a basin of hemispherical solar distiller ${Ex}_{out}$ calculated as^44^:

5 $${Ex}_{out}={h}_{e,w-g}\times {A}_{a}\times \left({T}_{bw}-{T}_{g}\right)\times \left(1-\frac{{T}_{a}}{{T}_{bw}}\right)$$

Coefficient of evaporative heat transfer ${h}_{e,w-g}$ calculated as^44^:

6 $${h}_{e,w-g}=16.273\times {10}^{-3}\times {h}_{c,w-g}\times \left(\frac{{P}_{bw}-{P}_{g}}{{T}_{bw}-{T}_{g}}\right)$$

Coefficient of convective heat transfer ${h}_{c,w-g}$ and the partial vapor pressure at free surface of basin water and the inner surface of glass P_bw_ and P_g_ are calculated by Eqs. (7–9)^44^:

7 $${h}_{c,w-g}=0.884\times {\left[\left({T}_{bw}-{T}_{g}\right)+\frac{\left({T}_{bw}+273.15\right)\times \left({P}_{bw}-{P}_{g}\right)}{\left(268900-{P}_{bw}\right)}\right]}^{\left(1/3\right)}$$

8 $${P}_{bw}=exp\left(25.317-\frac{5144}{\left({T}_{bw}+273.15\right)}\right)$$

9 $${P}_{g}=exp\left(25.317-\frac{5144}{\left({T}_{g}+273.15\right)}\right)$$

where; ${T}_{bw}, { T}_{a}, and {T}_{g}$ are the temperatures of water, ambient air, and glass cover (^o^C), respectively.

## Results and discussions

**Figure 4:**
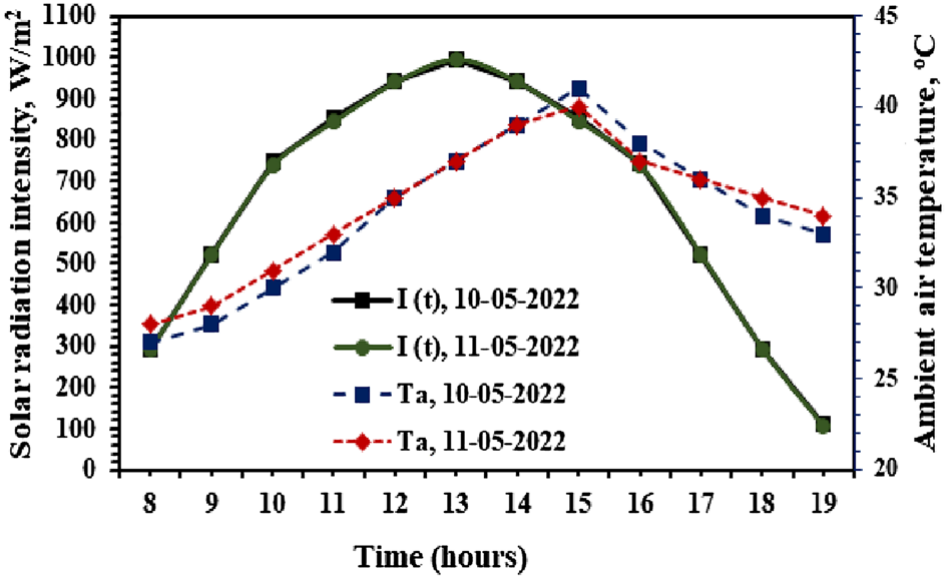
Measured solar radiation and surrounding air temperatures measured during the experiments.

The critical parameter in assessing the thermal performance of the solar still mainly depends on three factors, namely (i) ambient air temperature, (ii) wind velocity, and (iii) incident solar radiation. In these parameters, solar radiation is the total amount of heat input to drive the evaporation rate from the top liner of water from the circular basin of the hemispherical cover solar still. Moreover, the condensation of water vapor in the inner cover surface depends on the air temperature and wind velocity over the hemispherical cover. Therefore, to assess the thermal performance and exergy analysis from the SS using the proposed modification, the amount of solar radiation received and the surrounding air temperatures are measured every hour at the test location. Figure 4 shows the variations of both solar intensities received on the cover surface and the ambient air temperatures during the test period between 8:00 Hrs and 19:00 Hrs. It is observed from Fig. 4 that the solar incident radiation received on the horizontal plane of the SS reached a maximum of 995 and 990 W/m^2^ for test days 10th May, and 11th May, respectively. At the same time, the recorded ambient air temperatures at the test location varied between 27–41 and 38–40 °C for test days 10th May and 11th May, respectively.

**Figure 5:**
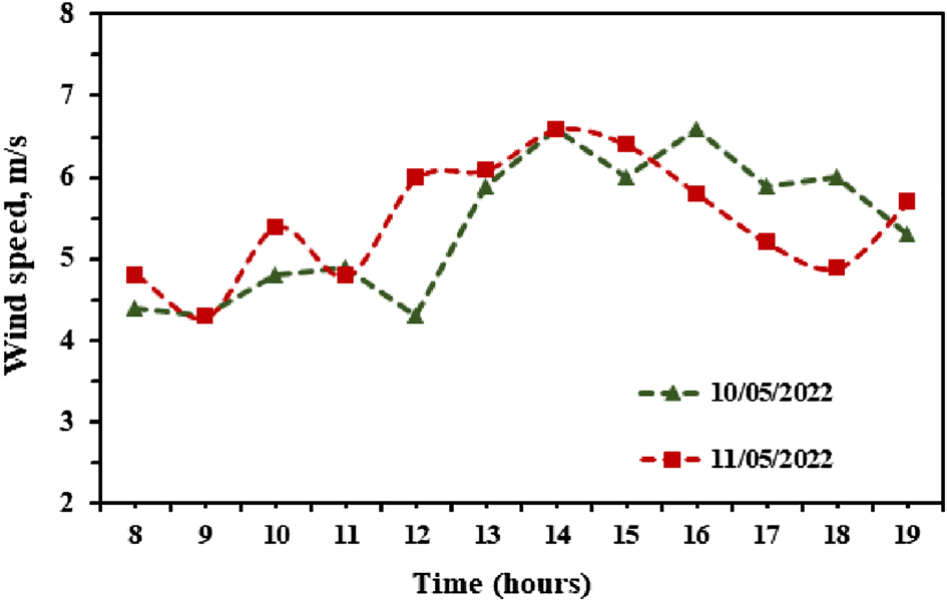
Measured wind speed during the experiments.

Figure 5 displays the variations of wind speed within two test days 10/05/2022 and 11/05/2022 starting from 8:00 am to 7:00 pm. As shown in Fig. 5 the wind speed ranges between 4.3 and 6.6 m/s during the test days.

**Figure 6:**
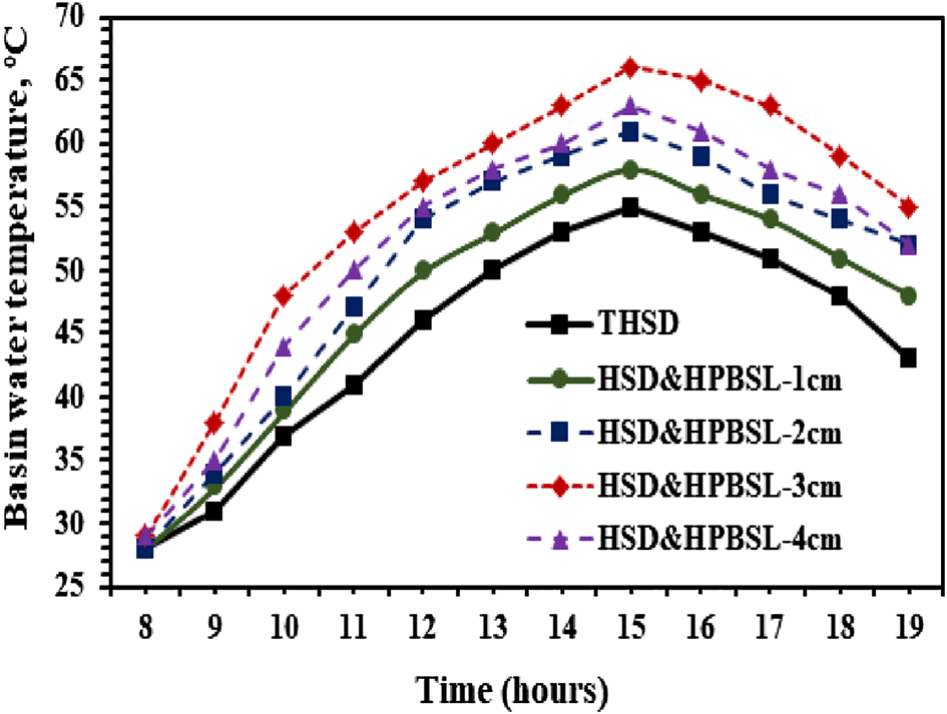
Basin water temperature variations from the HSS with and without high porosity black sponge layers.

**Figure 7:**
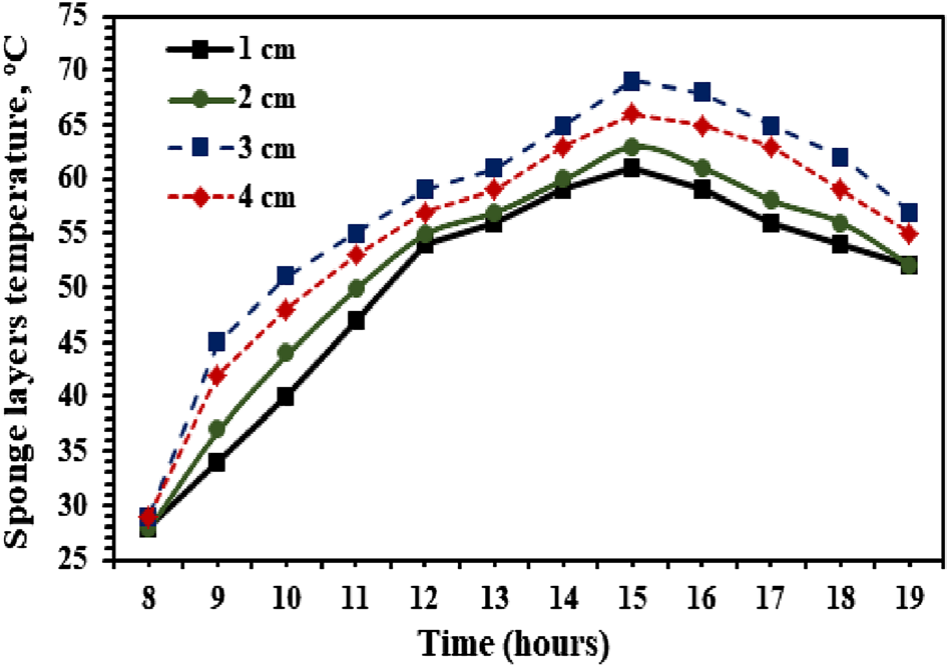
Variations of high porosity black sponge layers temperatures of hemispherical solar distillers.

**Figure 8:**
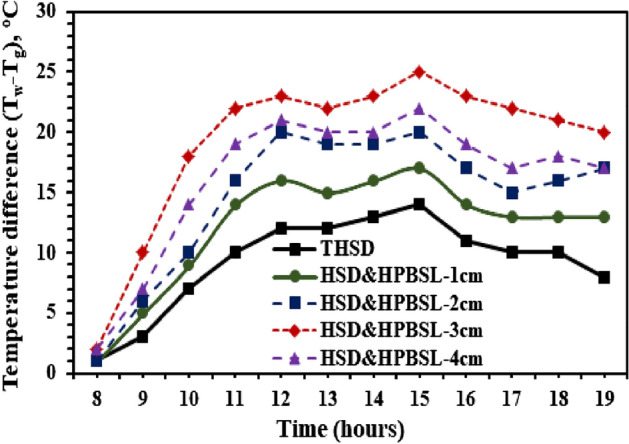
Difference in the temperature between the water and hemispherical cover from the HSS with and without high porosity black sponge layers.

To illustrate the influence of a black sponge high porous medium, the thickness of the black sponge on the thermal performance of HSD has to be optimized. The hourly changes in the water temperature, sponge layer thickness, and difference in temperature between the water and the hemispherical cover are depicted in Figs. 6, 7, 8, and the black sponge thickness is optimized during the test days between 8:00 Hrs and 19:00 Hrs.

Figure 6 shows the hourly variations of water temperature of hemispherical distillers with and without high porosity black sponge layers. It is clear from this that the basin water temperatures of a black sponge with higher porosity and increased thickness of sponge layers are enhanced compared to the HSS without utilizing the black sponge layer inside the still (THSD). The increment in the temperature of the water is proportional directly to the thickness of black sponge layers with higher porosity until they reach 3 cm. This is mainly due to the higher heat capacity of the black sponge layers, the porosity, and the capillary effect with the increase in thickness. After 30 mm, the improvement in the basin water temperature will be reduced, mainly because of the increment in the thermal resistance with a rise in the thickness of porous black sponge layers. The maximum recorded basin water temperatures reached 58, 61, 66, and 63 °C for utilizing the black sponge with higher porosity as layers with thicknesses of 10, 20, 30, and 40 mm, respectively, compared to 55 °C achieved for reference distiller THSD. It is clear that using a porous black sponge as an interfacial evaporation medium inside the basin of HSS improved the water temperature, and the optimum thickness of 3 cm inside the basin and increased porous thickness medium led to a decrease in the temperature of the water. This is evident from Fig. 7, where the maximum recorded temperatures of the high porosity black sponge layers reached 61, 63, 69, and 66 °C for the thickness of 10, 20, 30, and 40 mm, respectively.

**Figure 9:**
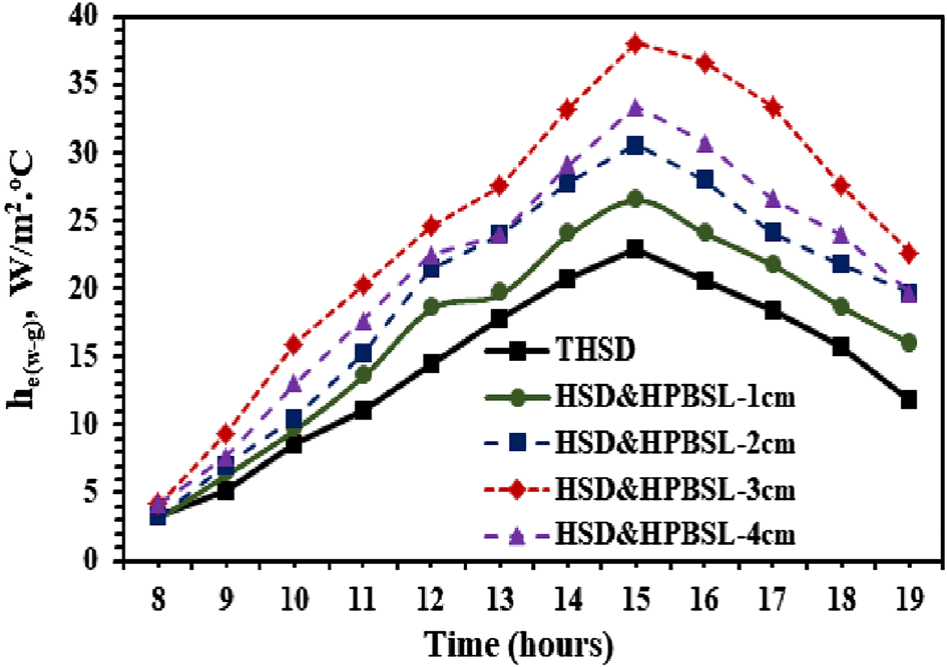
Evaporative heat transfer coefficient variations from the HSD with and without high porosity black sponge layers.

Figure 8 shows the variations of the temperature difference between water and cup for the hemispherical solar distillers with and without high porosity black sponge layers. It is clear from Fig. 8 that the temperature difference for utilizing the black sponge with higher porosity layers varies between 1–17, 1–20, 2–25, and 2–22 °C for HSD & HPBSL—1 cm, HSD & HPBSL—2 cm, HSD & HPBSL—3 cm, and HSD & HPBSL—4 cm, respectively, compared to 1–14 °C was achieved by the reference distiller (THSD). It is observed that using a 3 cm thickness black sponge as a highly porous medium inside the SS produced a maximum difference in temperature between the water and hemispherical cover, which occurred at 15:00 Hrs. This gives a good indication of the improvement in the evaporation rate in the case of using the black sponge with higher porosity layers with thicknesses of 3 cm (HSD & HPBSL—3 cm) compared to the reference distiller (THSD), and this is evident from Fig. 8, which shows the hourly variation of evaporative heat transfer between the basin water and hemispherical cover. From Fig. 9, it is seen that the evaporative heat transfer coefficient from the proposed modification (use of a black sponge as a porous medium) inside the hemispherical SS is higher as compared to the reference distiller (THSD). The coefficient of evaporative heat transfer varied between 3.22–22.93, 3.22–26.6, 3.22–30.56, 4.17–38.01, and 4.17–33.4 W/m^2 o^C for THSD, HSD & HPBSL—1 cm, HSD & HPBSL—2 cm, HSD & HPBSL—3 cm, and HSD & HPBSL—4 cm, respectively. These indicated that the maximum evaporative heat transfer was achieved by utilizing the high porosity black sponge layers with thicknesses of 30 mm (HSD & HPBSL—3 cm).

**Figure 10:**
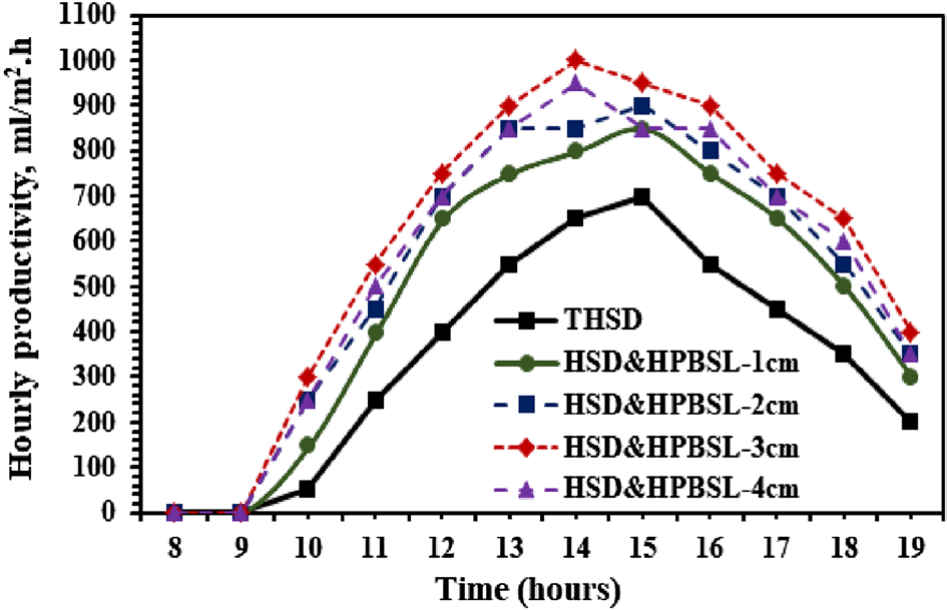
Hourly distillate water variations from the HSD with and without high porosity black sponge layers.

Figure 10 shows the hourly distillate water yield of hemispherical distillers with and without high porosity black sponge layers. From Fig. 10, it is clear that the hourly palatable water produced from the HSS using the black sponge as a porous medium is higher as compared to the HSD without a porous medium. These increments are proportional directly with the thickness of high-porosity black sponge layers until they reach 30 mm, mainly because of the increases in the evaporative heat transfer coefficients with an increase in the thickness of the high-porosity black sponge layers. After 3 cm, the improvement in the hourly distillate water yield will be reduced, mainly because of the decreases in the evaporative heat transfer coefficients with an increase in the thickness of high porosity black sponge layers after 30 mm. The peak distillate water collected from the HSS using the proposed modification reached up to 850, 900, 1000, and 950 mL/m^2^ h for utilizing the high porosity black sponge layers with 1, 2, 3, and 4 cm thicknesses, respectively, whereas, using a traditional HSD, the peak distillate collected was found as 700 mL/m^2^ h.

**Figure 11:**
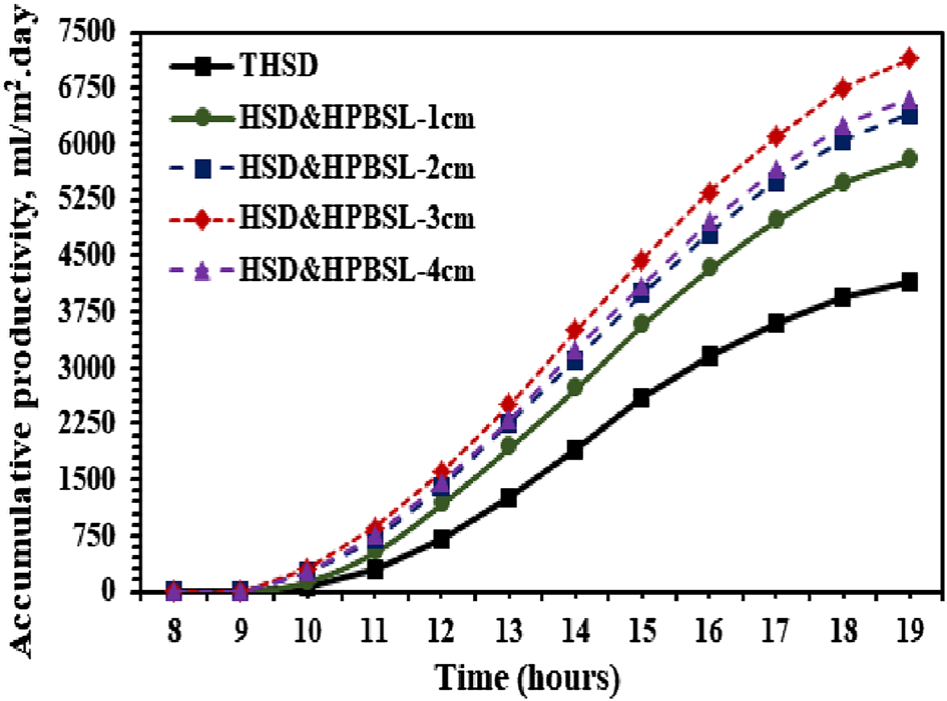
Daily palatable water from the HSD with and without high porosity black sponge layers.

The results indicate that using a black sponge as a porous medium inside the SS improved the evaporation rate and, thereby, cumulative yield. It was also found that the optimum thickness of the black sponge was 3 cm, which produced the maximum hourly yield compared to traditional HSD and HPBSL—3 cm and decreased on the increased thickness of the black sponge layer as a porous medium. The cumulative potable water produced from the SS using the proposed modification (use of a black sponge as the porous medium) is depicted in Fig. 11. It is seen that the hemispherical distiller with 1, 2, 3, and 4 cm of black sponge as the porous medium produced total potable water of about 5800, 6400, 7150, and 6600 mL/m^2^ respectively, whereas, the traditional HSD produced maximum potable water of 4150 mL/m^2^. Similarly, the corresponding augmentation on the potable water from the SS using the black sponge as the high porous medium was found as 39.76, 54.22, 72.29, and 59% for thicknesses of 1, 2, 3, and 4 cm, respectively.

**Figure 12:**
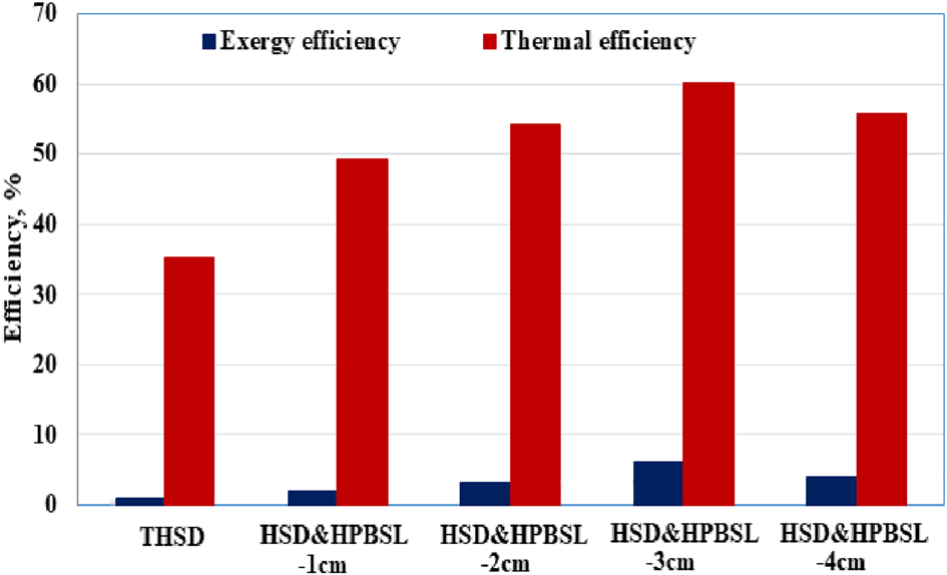
Daily exergy and thermal efficiencies of the various proposed hemispherical distillers.

The influence of utilizing the high porosity black sponge layers on the thermal and exergy efficiencies is shown in Fig. 12. It is clear that the influence of black sponge as a porous medium inside the hemispherical solar distiller improved the daily thermal efficiency to 49.24, 54.19, 60.25, and 55.8% for 1, 2, 3 and 4 cm as thickness of black sponge respectively and it was higher as compared to the traditional HSD (35.33%). The use of the high porosity black sponge layers increased the exergy efficiency from 1.01% for the reference distiller (THSD) case to 1.92, 3.13, 6.19, and 3.94% for thicknesses of 1, 2, 3, and 4 cm, respectively.

Table 2 shows the summary of the effect of using high-porosity black sponge layers on the accumulative productivity and thermal and exergy efficiencies of hemispherical distillers. As shown in this table, the utilization of the high porosity black sponge layer with a thickness of 3 cm (HSD & HPBSL—3 cm) produced the highest thermal and exergy efficiency from the hemispherical solar distiller. It was found that using the high porosity black sponge layers with thicknesses of 30 mm (HSD & HPBSL—3 cm) improved the accumulative distillate yield from 4150 mL/m^2^ day for THSD case to 7150 mL/m^2^ day with an improvement of 72.29%. Also, it was found that using the high porosity black sponge layers with thicknesses of 30 mm (HSD & HPBSL—3 cm) improved the thermal efficiency from 35.33% for the reference distiller (THSD) case to 60.25% with an improvement of 70.53%. Moreover, using the high porosity black sponge layers with thicknesses of 30 mm (HSD & HPBSL—3 cm) improved the exergy efficiency from 1.01% for the reference distiller (THSD) case to 6.19% with an improvement of 512.87%.

**Table 2:** Summary of the productivity, exergy, gain in exergy, and energy and gain in energy efficiencies of HSD using different thicknesses of black sponge porous medium.

	THSD	HSD&HPBSL			
		1 cm	2 cm	3 cm	4 cm
Accumulative productivity, l/m^2^ day	4.15	5.8	6.4	7.15	6.6
Gain in productivity, %	–	39.76	54.22	72.29	59
Thermal efficiency, %	35.33	49.24	54.19	60.25	55.8
Gain in thermal efficiency, %	–	39.37	53.38	70.53	57.94
Exergy efficiency, %	1.01	1.92	3.13	6.19	3.94
Gain in exergy efficiency, %	–	90.1	209.9	512.87	290.1

## Economic analysis

The economic analysis of our current system was investigated to show the economic feasibility of using the high-porosity black sponge layers and their impact on the price of a liter of water produced from solar distillers. The calculations for economic feasibility were carried out using the steps described by^45^. The breakdown of the cost involved in the evaluation of the cost of distilled water produced is tabulated in Table 3, and it is clear that using a 3 cm thick high porosity black sponge layers (HSD & HPBSL—3 cm) achieved the lowest cost per liter produced from hemispherical solar distillers, which was estimated at about 0.007 $/l compared to 0.012 $/l achieved by the reference distiller (THSD). Therefore, the use of a high porosity black sponge layer with a thickness of 3 cm (HSD & HPBSL—3 cm) represents an effective option as it reduced the cost of distillate water per liter produced from hemispherical solar distillers by 41.67% compared to the reference distiller (THSD).

**Table 3:** Economic analysis of HSD with and without black sponge porous medium as the interfacial evaporation medium.

	THSD	HSD & HPBSL			
		1 cm	2 cm	3 cm	4 cm
Total capital cost, $	61.55	61.96	62.17	62.37	62.58
Annual capital cost, $	10.89	10.96	11	11.04	11.08
Annual salvage value, $	0.7	0.71	0.71	0.71	0.71
Annual operating and maintenance cost, $	3.27	3.29	3.3	3.31	3.32
Annual total cost, $	13.46	13.54	13.59	13.64	13.69
Annual distillate productivity, l/m^2^.year	1121	1566	1728	1931	1782
Water cost per liter, $/l	0.012	0.0086	0.0078	0.007	0.0076
Reduced cost of distilled water productivity compared to THSD, %	–	28.33	35	41.67	36.67

## Conclusion and future recommendation

The current work aims to improve the thermal performance and efficiency of the hemispherical cover solar distiller (HSD). The HSD has a greater advantage over the other traditional single slope solar distillers as the contact surface area hemispherical cover is exposed to the wind velocity and ambient interaction. The lower cover temperature leads to a higher rate of condensation. Therefore, the increase in the rates of evaporation inside the HSD represents an important axis to achieve the maximum benefit from the low temperature of the hemispherical cover and the increase in the productivity of the HSD from distilled water. To achieve this goal, a high porosity black sponge layer for improved interfacial evaporation was used to achieve the highest rates of evaporation and condensation within the hemispherical cover solar distillers. To get the optimal thickness of the porous sponge layers, the different thicknesses of porous sponge layers varied from 1 to 4 cm were used in the hemispherical distiller, studied, and compared to the reference HSD without porous sponge medium. The main results from the experiments are concluded below:

- Using the high porosity black sponge layers increased the daily accumulative distillate productivity from 4150 mL/m^2^ for conventional HSD cases to 5800, 6400, 7150, and 6600 mL/m^2^ for thicknesses of 1 cm, 2 cm, 3 cm, and 4 cm, respectively.
- The improvement in accumulative distillate productivity for utilization of high porosity black sponge layers reached 39.76, 54.22, 72.29, and 59% for the thicknesses of 1 cm, 2 cm, 3 cm, and 4 cm, respectively.
- Using the high porosity black sponge layers increased the thermal efficiency from 35.33% for conventional HSD case to 49.24, 54.19, 60.25, and 55.8% for the thicknesses of 1 cm, 2 cm, 3 cm, and 4 cm, respectively.
- Using the high porosity black sponge layers increased the exergy efficiency from 1.01% for conventional HSD case to 1.92, 3.13, 6.19, and 3.94% for the thicknesses of 1 cm, 2 cm, 3 cm, and 4 cm, respectively.
- Using the high porosity black sponge layer with a thickness of 3 cm (HSD & HPBSL—3 cm) represents an effective option as it reduced the cost per liter produced from the hemispherical solar distillers by 41.67% compared to conventional HSD.

In the future, the Authors recommended a comprehensive comparison study to obtain the best design configurations and materials of the porous mediums that achieve the highest evaporation rates, in addition to studying the influences of added nanoparticles to porous materials on the yield of the hemispherical cover solar distiller.

## Data Availability

The datasets used and/or analysed during the current study available from the corresponding author on reasonable request.

## List of symbols

$${A}_{a}$$: Absorber area, m^2^
${h}_{fg}$: Latent heat, J/kg
I(t): Solar radiation, W/m^2^
${\dot{m}}_{dw}$: Distillate yield, kg/h
${T}_{a}$: Ambient air temperature, °C
${\mathrm{T}}_{bw}$: Basin water temperature, °C
${T}_{g}$: Glass cover temperature, °C
$${\eta }_{ex.}$$: Exergy efficiency, %
$${\eta }_{th}$$: Thermal efficiency, %

## Abbreviations

HSD: Hemispherical solar distiller
HSD & HPBSL: Hemispherical solar distillers contain high porosity black sponge layers
THSD: Traditional hemispherical solar distiller
